# Anxiety among patients with single versus multiple visits for root canal treatment

**DOI:** 10.6026/973206300211563

**Published:** 2025-06-30

**Authors:** Saicharan G., Joel Mathew, Renjith Madhavan, Ajeesha Nair J.S, Sonam Dubey, Udipta Sahoo

**Affiliations:** 1 Department of Conservative Dentistry & Endodontics, Mahe Institute of Dental Sciences & Hospital, Chalakkara, Pallor, Mahe - 673 310 U.T of Puducherry, Mahe, India; 2 Department of Paediatric and Preventive Dentistry, Mar Baselios Dental College, Thankalam P.O, Kothamangalam, Kerala -686691, India; 3 Department of Periodontology, Century International Institute of Dental Sciences and Research Center, Poinachi, Thekkil Kasaragod - 671541 Kerala, India; 4 Department of Orthodontics and Dentofacial Orthopaedics, Century International Institute of Dental Sciences and Research Center, Poinachi Thekkil Kasaragod - 671541 Kerala, India; 5 Department of Conservative Dentistry and Endodontics, Senior resident at Graphic Era institute of medical science, Dehradun, Uttarakhand, India; 6 Department of dentistry, Kalinga Institute of Dental Sciences, KIIT Deemed to be University, Bhubaneswar, India

**Keywords:** Single-visit root canal therapy, Multiple-visit RCT, patient anxiety, irreversible pulpitis, endodontic outcomes, pain assessment, treatment success rate

## Abstract

RCT treatment makes patients feel nervous often and the option to do the procedure in one session or divided across multiple visits affects both patients' peace of mind during the process and the treatment results. The investigators split 60 individuals with irreversible pulpitis into two groups that received RCT as one continuous session or performed across several visits. Patients who had single-visit treatment experienced greater anxiety decrease from 18.6 points to 11.2 points whereas patients receiving multiple sessions showed less significant anxiety reduction from 19.1 points to 13.7 points. Single-visit RCT led to lower pain scores in patients compared to multiple-visit RCT at one week yet success rates between both groups remained similar at 93% versus 90% (p>0.05). Patient anxiety improved better after single-visit RCT procedures despite achieving comparable treatment outcomes.

## Background:

Root canal treatment (RCT) remains the cornerstone of managing irreversible pulpitis and periapical pathologies. Although RCT is highly successful, patients frequently report significant anxiety due to misconceptions about pain, treatment complexity, and number of sessions [1, 2]. Dental anxiety not only impacts patient cooperation but also contributes to appointment avoidance and compromised clinical outcomes [2]. The decision between single-visit and multiple-visit RCT continues to be a clinical dilemma. Proponents of single-visit RCT argue it reduces patient burden, eliminates inter-appointment contamination, and enhances compliance by completing the procedure in one setting [3, 5 and 7]. Conversely, multiple-visit RCT is preferred in cases with necrotic pulps or persistent exudate, where intra-canal medicaments such as calcium hydroxide are used to reduce microbial load [4, 9]. However, the effectiveness of calcium hydroxide in completely eradicating intra-radicular infection has been challenged. Studies show no significant difference in periapical healing or bacterial reduction between single and multiple visits, especially when contemporary instrumentation and irrigation protocols (*e.g.*, rotary files, NaOCl, and CHX) are employed [6, 7 and 10]. Pain outcomes remain equivocal. Some investigations found no difference in postoperative discomfort between the two approaches [6], while others reported reduced pain in single-visit RCT [5, 12]. Likewise, overall success rates are comparable, with healing and functional outcomes remaining statistically similar [3, 10 and 11]. Psychological aspects of RCT-particularly patient anxiety-have received limited attention in comparative studies. Evidence suggests that prolonged treatment intervals may exacerbate anxiety and pain anticipation, negatively influencing patient satisfaction and perception of care [12, 13]. Shorter treatment durations, when clinically appropriate, may therefore yield higher patient acceptability without compromising success [11, 14]. Furthermore, newer diagnostic tools like cone-beam computed tomography (CBCT) improve lesion detection and procedural planning, which can support the efficacy of single-visit treatment in selected cases [8]. As patient-centered care gains prominence, understanding the psychological and clinical outcomes of treatment schedules is essential. Therefore, it is of interest to compare the effect of single-visit versus multiple-visit RCT on dental anxiety and clinical outcomes, including pain and treatment success. By utilizing the Modified Dental Anxiety Scale (MDAS) and clinical follow-ups, we assess how treatment modality influences both psychological comfort and therapeutic efficacy in patients with irreversible pulpitis.

## Materials and Methods:

A total of 60 patients received treatment in this randomized clinical research which focused on single-rooted teeth with irreversible pulpitis. All participants gave their informed consent after which the study received approval from the institutional review board. The research enrolled patients who displayed irreversible pulpitis together with minimal periapical involvement or solely irreversible pulpitis. The study included patients whose age fell between 18 and 50 years old with good general health before their teeth required root canal treatment. Patients who did not have systemic diseases or a previous history of treatments on the tooth also qualified. The research study excluded patients with teeth displaying acute abscesses, swelling, periodontal pockets beyond 4 mm or a previous 48-hour intake of antibiotics or analgesics.

Participants were randomly divided into two groups using a computer-generated randomization table:

[1] Group A (Single-Visit RCT, n = 30): Root canal treatment was completed in a single appointment.

[2] Group B (Multiple-Visit RCT, n = 30): Treatment was carried out over two appointments, with calcium hydroxide as an intracanal medicament between visits.

A solitary endodontist performed all procedures to guarantee procedural uniformity. A local anesthetic consisting of 2% Lidocaine with 1:80,000 epinephrines served as the anesthesia method. Every procedure received rubber dam isolation as part of the standard protocol. The dental professionals used standard procedures to prepare the access cavities and determined the working lengths through apex locators and subsequent radiographic verification. The ProTaper Universal system files provided canal instrumentation during which time 2.5% sodium hypochlorite and saline acted as irrigation agents. The multiple-visit group received treatment with calcium hydroxide as a medicament in addition to sealing the canals temporarily with Cavit. The restoration technicians carried out additional irrigation followed by filling procedures when they visited the canal for a second time seven days after the initial visit. All tooth canal obturations occurred during the same appointment through the application of gutta-percha and AH Plus sealer with cold lateral compaction technique. The dentist applied composite resin as the final restoration method. The Modified Dental Anxiety Scale (MDAS) was used to measure patient anxiety before and right after treatment execution. One-week post-operative pain evaluation happened by means of Visual Analog Scale (VAS) scores. The evaluation of clinical success required periapical radiographs taken at 3 months which checked for both symptom absence and periapical healing. The statistical analysis occurred with version 26 of SPSS software. Analysis of changes in anxiety scores utilized paired t-tests whereas success rates between groups were compared through chi-square tests. The study considered statistical significance at a p-value level below 0.05.

## Results:

A total of 60 patients completed the study, with 30 in each group. The demographic distribution was comparable between both groups, with no statistically significant difference in age or gender (mean age: Group A = 34.5 ± 8.2 years; Group B = 33.9 ± 7.6 years; p = 0.72).

## Patient anxiety scores:

The Modified Dental Anxiety Scale (MDAS) scores showed a significant reduction in both groups post-treatment. Group A (single-visit) demonstrated a greater decrease in anxiety compared to Group B (multiple-visit). As shown in Table 1 (see PDF), both groups experienced a statistically significant reduction in anxiety levels, but the reduction was greater in the single-visit group (p < 0.001).

## Postoperative pain (VAS Scores):

Pain intensity was evaluated using the Visual Analog Scale (VAS) at 1-week post-treatment. Table 2 (see PDF) indicates that patients in Group A reported significantly less postoperative pain at 1-week follow-up compared to Group B (p = 0.045). Indicates that patients in Group A reported significantly less postoperative pain at 1-week follow-up compared to Group B (p = 0.045).

## Clinical success rate:

Clinical and radiographic evaluations were conducted at the 3-month follow-up to assess treatment success. As illustrated in Table 3 (see PDF), the clinical success rate was slightly higher in the single-visit group (93%) compared to the multiple-visit group (90%), though the difference was not statistically significant (p = 0.64). In summary, single-visit RCT showed better outcomes in terms of anxiety reduction and pain relief, while both treatment protocols demonstrated comparable success rates in periapical healing (Table 1 - see PDF, Table 2 - see PDF and Table 3 - see PDF).

## Discussion:

This study investigated the psychological and clinical effects of single-visit versus multiple-visit root canal therapy (RCT) on patients diagnosed with irreversible pulpitis. The findings suggest that while both treatment modalities achieved comparable clinical success, the single-visit group experienced significantly greater reduction in dental anxiety and postoperative pain-indicating potential patient-centered advantages. The observed reduction in anxiety among patients undergoing single-visit RCT is consistent with findings by Wong *et al.* [1, 2], who emphasized that procedural simplicity and fewer appointments can improve patient perception and reduce stress. Furthermore, Paredes-Vieyra *et al.* [3] and Eyuboglu *et al.* [4] reported that patients undergoing fewer treatment sessions often show improved satisfaction, which supports our result that single-visit RCT can alleviate psychological discomfort more effectively. Pain outcomes in our study also favored single-visit therapy, with patients reporting lower postoperative discomfort at one week. This aligns with results from Edionwe *et al.* [5] and Saoud *et al.* [7], who noted reduced pain in single-session RCT, potentially due to minimized inter-appointment contamination and reduced risk of temporary restoration failure, as also discussed by de Paula-Silva *et al.* [8]. In contrast, Figini *et al.* [6], in a Cochrane systematic review, found no significant difference in postoperative pain between single- and multiple-visit RCT. This discrepancy may stem from variation in case selection, tooth type, instrumentation techniques, and operator expertise. Similarly, Prashanth *et al.* [9] and Koch [10] argued that postoperative pain is more closely linked to preoperative status and canal morphology than the number of treatment sessions, which may explain variability across the literature. Regarding treatment outcomes, our results show that clinical success-measured by the absence of pain and radiographic healing-were comparable in both groups. This observation aligns with the conclusions of Fonzar *et al.* [11], Vera *et al.* [12], and Olcay *et al.* [13], who found no statistically significant differences in long-term success rates between single- and multiple-visit RCT. The use of modern rotary instrumentation and effective irrigation agents in both groups may explain the similar success rates in our study and those previous studies. Although calcium hydroxide was used as an intracanal medicament in the multiple-visit group, the overall success rate remained comparable to the single-visit group. Vera *et al.* [12] and Penesis *et al.* [15] highlighted that calcium hydroxide, while antimicrobial, does not ensure complete bacterial elimination. Furthermore, Lin *et al.* [14] reported that calcium hydroxide might be less effective in vital pulp cases with minimal periapical involvement, suggesting that in such scenarios, single-visit treatment may suffice. Despite positive outcomes, several limitations should be acknowledged. This study focused on single-rooted teeth with irreversible pulpitis, limiting its generalizability to multi-rooted teeth or necrotic cases. Additionally, the three-month follow-up period may not detect late failures; hence, longer observation is needed to validate our findings.

## Conclusion:

We show that single-visit RCT proves beneficial for appropriate tooth conditions specifically when patients require decreased anxiety and efficient appointment planning. Clinical outcomes between two treatment approaches were equivalent though single-session treatment brought the advantage of patient comfort and enhanced patient satisfaction.
